# Analysis of Carbon Density Influencing Factors and Ecological Effects of Green Space Planning in Dongjiakou Port Area

**DOI:** 10.3390/plants14142145

**Published:** 2025-07-11

**Authors:** Yuanhao Guo, Yaou Ji, Qianqian Sheng, Cheng Zhang, Ning Feng, Guodong Xu, Dexing Ma, Qingling Yin, Yingdong Yuan, Zunling Zhu

**Affiliations:** 1 College of Landscape Architecture, Nanjing Forestry University, Nanjing 210037, China; guoyuanhao@njfu.edu.cn (Y.G.); jiyaou@njfu.edu.cn (Y.J.); zhangcheng@njfu.edu.cn (C.Z.); zhuzunling@njfu.edu.cn (Z.Z.); 2 Co-Innovation Center for the Sustainable Forestry in Southern China, Nanjing Forestry University, Nanjing 210037, China; 3 Jin Pu Research Institute, Nanjing Forestry University, Nanjing 210037, China; 4 Research Center for Digital Innovation Design, Nanjing Forestry University, Nanjing 210037, China; 5 Qingdao Municipal Engineering Design and Research Institute Co., Ltd., Qingdao 266000, China; 6 College of Art and Design, Nanjing Forestry University, Nanjing 210037, China

**Keywords:** port greening, carbon storage, urban planning and development

## Abstract

Port green spaces are essential protective barriers, enhancing safety and environmental resilience in high-activity port regions. Given the intensity of human activities in these areas, understanding the factors influencing the carbon sequestration capacity and ecological benefits of port green spaces is crucial for developing sustainable green ports. This study integrated field investigations and remote sensing data to estimate carbon density and carbon sequestration capacity in the Dongjiakou Port area, examining their relationship with port green space planning. The results indicated that carbon density in green spaces showed a significant negative correlation with the number of lanes in adjacent roads, where an increase in lane numbers corresponded to lower carbon density. Additionally, carbon density decreased significantly with increasing distance from the shipping center. In contrast, a significant positive correlation was observed between carbon density and distance from large water bodies, indicating that green spaces closer to large water bodies exhibited smaller carbon density. Infrastructure development in Dongjiakou substantially negatively impacted vegetation carbon sequestration capacity, with effects not reversible in the short term. However, green space enhancement efforts provided additional ecological benefits, leading to a 50.9 ha increase in green space area. When assessing carbon density in urbanizing areas, geographical influences should be prioritized. Furthermore, the long-term environmental impacts of urban expansion must be considered at the early planning stages, ensuring the implementation of proactive protective measures to mitigate potential ecological disruptions.

## 1. Introduction

Over the past century, CO_2_ emissions from human activities have been the primary driver of recent climate change [1]. Reducing carbon emissions and enhancing plant-based carbon sequestration are recognized as effective strategies for mitigating atmospheric CO_2_ accumulation [2,3]. Rapid urbanization and increasing resource demand have directly or indirectly disrupted ecosystems. High population density, industrial activities, and transportation systems have contributed to significant air pollutants and greenhouse gas emissions [4], leading to structural and functional imbalances in urban ecosystems that exceed their carrying capacity [5]. Urban vegetation offers nature-based solutions to various environmental challenges [6,7], playing a crucial role in mitigating the urban heat island effect, improving air quality, and promoting carbon neutrality. Understanding the impact of human activities on urban ecosystems remains essential.

Carbon sequestration in urban ecosystems is influenced by both natural and anthropogenic factors. The mechanisms governing urban carbon sinks are significantly more complex than those in single ecosystems, requiring more detailed investigations into the relationship between urbanization and carbon sequestration [8]. Vegetation characteristics such as tree age, community density, and canopy closure strongly affect carbon sequestration capacity [9,10,11]. While extensive research has been conducted in this area, most studies have focused on forest ecosystems. In the context of anthropogenic influences within urban ecosystems, Xu et al. [12] found that the locations of city centers, roads, rivers, and railways can impact plant-based carbon sequestration. Liu [13] analyzed carbon storage across different regions and observed significant spatial heterogeneity, with higher aboveground carbon storage in rural areas than in urban areas. Research also indicates that managed green spaces with frequent maintenance exhibit higher carbon sequestration capacity, partially offsetting emissions from urban development [14]. The complexity of ecological factors affecting carbon sequestration in urban ecosystems increases uncertainties in understanding their carbon sink potential.

Urban park vegetation communities and spatial configurations play a crucial role in the surrounding environment, directly affecting residents [15,16]. The ecological value of green spaces in urban cores and carbon emissions from heavily polluted areas [17] often receive significant attention. Vegetation near heavily polluted and industrial areas is often overlooked. Despite being planted extensively and possessing stronger stress resistance to serve as isolation and protective barriers, it is also more susceptible to environmental influences. The role and impact of such green spaces in carbon sequestration under human activities should not be neglected. Many researchers have utilized field survey data at various resolutions or remote sensing imagery [18] and applied multivariate statistical analyses to examine the relationship between urban green space carbon sequestration and ecological factors such as green space area, canopy closure, vegetation diversity, and vegetation indices [19,20]. Although numerous remote sensing vegetation indices have been developed to capture vegetation structure and function, many exhibit limitations in regional adaptability, computational efficiency, or robustness under heterogeneous environmental conditions. These constraints reduce their effectiveness in large-scale or site-specific carbon estimation. Therefore, selecting indices with high generalizability and operational feasibility is essential for improving the accuracy and applicability of remote sensing-based inversion models [21,22]. Large-scale field surveys require substantial resources, whereas remote sensing provides rapid access to extensive urban green space data but lacks detailed spatial information [23]. Integrating both methods enhances the efficiency and accuracy of green space assessments. Most studies conduct field measurements of vegetation parameters such as diameter at breast height (DBH) and tree height, estimate biomass using allometric equations, and convert biomass into carbon storage. Remote sensing-derived vegetation indices are combined with environmental variables and carbon storage data to develop multiple regression models for estimating carbon density or total carbon storage in specific areas [24,25,26].

The primary objective of this study was to assess the carbon sequestration capacity of green spaces in the Dongjiakou Port area and analyze the interactions between anthropogenic factors and port green spaces. Field surveys were conducted on various green spaces established during the same period, examining differences in vegetation characteristics and the varying impacts of human activities over time. The findings contribute to a better understanding of the interplay between port green spaces and anthropogenic influences, providing insights for improving urban ecological quality.

## 2. Materials and Methods

### 2.1. Study Area Overview

**Figure 1:**
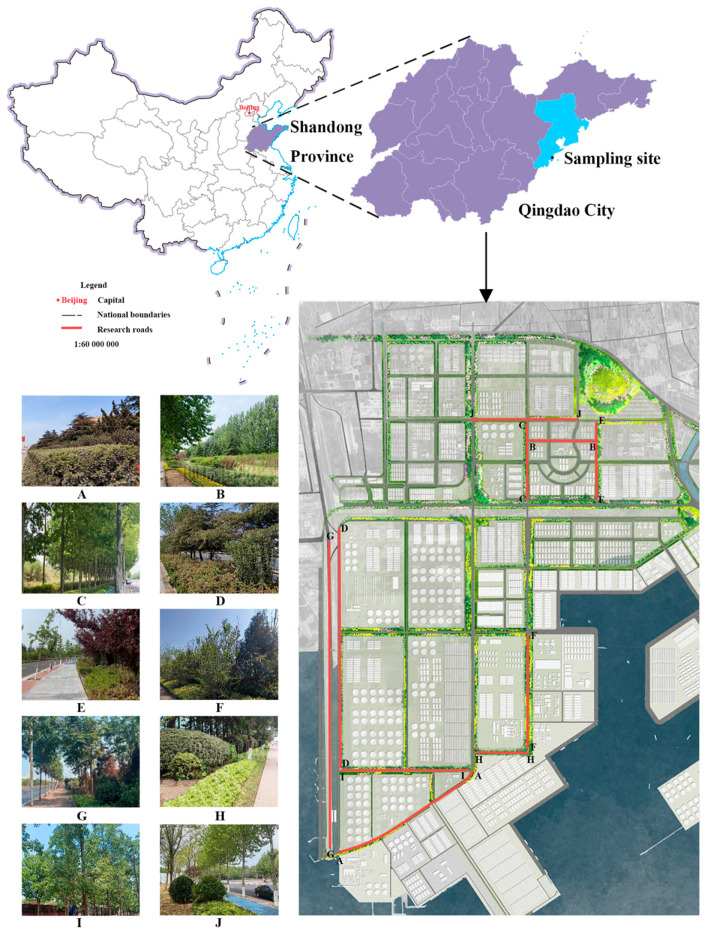
Location map of the Dongjiakou port area in Qingdao. Note: (**A**) Banan Road; (**B**) Wei’er Road; (**C**) Jing’er Road; (**D**) Jingshi Road; (**E**) Jingwu Road; (**F**) Jingyi Road; (**G**) Shugangyi Road; (**H**) Weishisi Road; (**I**) Weishiwu Road; (**J**) Weiyi Road.

This study was conducted in the Dongjiakou Port area of Qingdao Port (119°42′26.02″–119°48′5.32″ E, 35°36′18.43″–35°39′42.30″ N) (Figure 1). Located in the southern part of Qingdao, the port covers a planned total area of 70 km^2^. Influenced by a marine climate, the region experiences mild and humid conditions, with no severe cold in winter and no extreme heat in summer. The average annual temperature is 12.2 °C. Precipitation is concentrated between June and September, accounting for 71.4% of the annual total, with an average annual rainfall of 662.1 mm. The initial planning and construction of the Dongjiakou Port area began in 2009, with partial roadside greening carried out in 2021. In 2024, further upgrades and enhancements to green spaces were implemented. All surveyed green spaces in this study were established during the same construction period.

### 2.2. Data Sources

**Figure 2:**
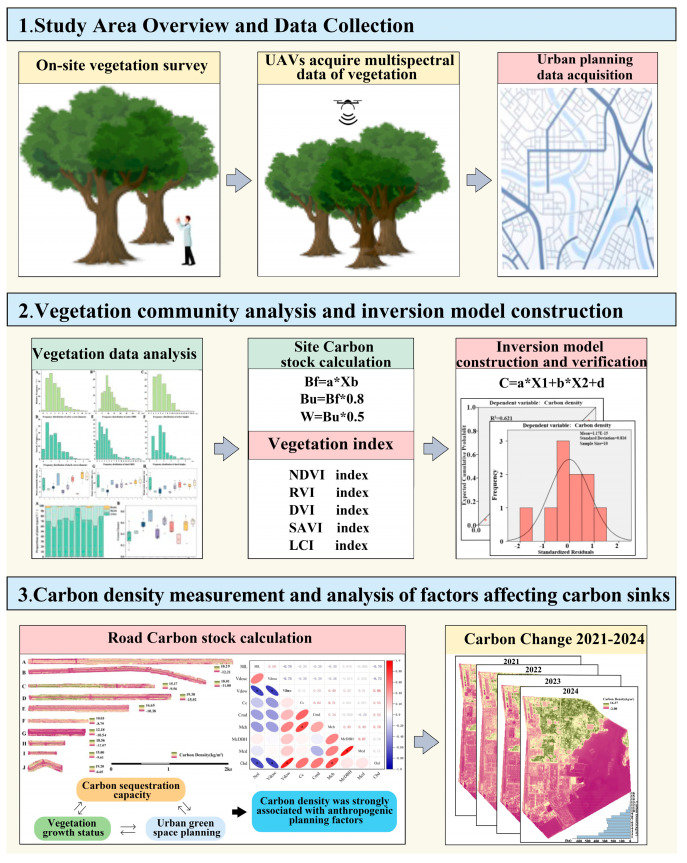
Research flow chart.

A field survey was conducted in the Dongjiakou Port area from 12–13 June 2024, focusing primarily on roadside green spaces. The total area of green space involved in this study was 2887.01 hectares. Due to real-time changes in the actual area of green spaces, the data measured on 30 May 2024 were used. The surveyed road sections included Weiyi Road, Wei’er Road, Jing’er Road, Jingwu Road, Shugangyi Road, Weishiwu Road, Banan Road, Weishisi Road, Jingshi Road, and Jingyi Road. Vegetation data were collected through an integrated stratified and systematic sampling approach to ensure both ecological representativeness and spatial uniformity. The study area was initially stratified based on road segments, vegetation types, and canopy structure to capture the heterogeneity of the green spaces. Subsequently, a 100 m regular grid was established centered on the port area, with grid centroids serving as candidate sampling units. After excluding invalid points, samples were systematically ranked and selected at equal intervals through statistical methods, resulting in a total of 60 sampling plots. This sampling design facilitated an even spatial distribution of sampling locations, enhanced the efficiency of field data acquisition, minimized spatial autocorrelation and sampling bias, and improved the precision of models based on remote sensing inversion methods [27]. Field data collection involved measuring tree species, diameter at breast height (DBH), canopy width, canopy closure, green space type, and vegetation community type for different plant species at 60 sample plots along 10 road sections. Measurement tools included a diameter tape and marking pens (Figure 2).

Remote sensing techniques were used to extract green space information for the port area. Urban green space boundaries were delineated by overlaying the overall urban planning framework with the regional green space system plan. A DJI Phantom 4 Multispectral drone(Nanjing, China) was used to acquire multispectral imagery along the 10 surveyed roads at a flight altitude of 103 m and a speed of 6 m/s. Multispectral data from 2021 to 2024 were obtained from Landsat 8, while green space planning data for Dongjiakou were sourced from the Qingdao Public Resource Transaction Electronic Service System [28].

### 2.3. Data Processing

#### 2.3.1. Estimation of Carbon Storage and Vegetation Community Characteristics in Sample Plots

Vegetation data, including tree height, canopy width, and DBH, were obtained through field surveys of urban green spaces. This study focused on urban green spaces where vegetation was sparsely distributed and plant density was relatively low. Compared to forest ecosystems, urban ecological systems are more structurally and functionally complex. Given the objective of conducting large-scale carbon estimation and supporting remote sensing-based inversion analysis—along with the fact that organs such as leaves in urban plant communities are more susceptible to environmental influences—this study adopted a direct estimation of total aboveground biomass for individual trees. Biomass for individual trees within the sample plots was estimated using allometric biomass equations. Since most existing allometric equations were developed for forest ecosystems, a conversion coefficient of 0.8 was applied to adapt calculations for urban green spaces. Carbon storage in each sample plot was then estimated based on biomass and a carbon content ratio of 0.5 [29]. The allometric biomass equations used in this study were referenced from the existing literature (Table 1). When species-specific equations were unavailable, broadleaf and conifer equations were applied accordingly. Final carbon storage estimates for each sample plot were obtained by aggregating individual tree calculations.

Vegetation community characteristics, including average tree height, canopy width, and DBH, were calculated using a weighted approach based on the biomass of trees and shrubs within the community [30].

B_f_ = a × X^b^ (1)

where B_f_ represents forest biomass; X is a measured plant variable, including DBH (D), canopy width (C), projected canopy area (AC = πC1C2/4), and tree height (H); and a and b are species-specific parameters.

B_u_ = B_f_ × 0.8 (2)

where B_u_ represents urban biomass.

W = B_u_ × 0.5 (3)

where W is the carbon storage.

Mch = B_fa_/(B_fa_ + B_fs_) × Mch_a_ + B_fs_/(B_fa_ + B_fs_) × Mch_s_ (4)

where Mch represents the average tree height of the vegetation community; B_fa_ and B_fs_ are the biomass of trees and shrubs, respectively; and Mch_a_ and Mch_s_ denote the average tree height of trees and shrubs.

McDBH = B_fa_/(B_fa_ + B_fs_) × McDBH_a_ + B_fs_/(B_fa_ + B_fs_) × McDBH_s_ (5)

where McDBH represents the average DBH of the vegetation community and McDBH_a_ and McDBH_s_ are the average DBH of trees and shrubs, respectively.

Mcd = B_fa_/(B_fa_ + B_fs_) × Mcd _a_ + B_fs_/(B_fa_ + B_fs_) × Mcd _s_ (6)

where Mcd represents the average canopy width of the vegetation community, while Mcd_a_ and Mcd_s_ denote the average canopy width of trees and shrubs, respectively.

**Table 1:** Plant biomass equations.

Tree Species	Plant Biomass Equations	Sources
*Pinus tabuliformis*	B = 0.8446 + 0.6640ln(D^2^H)	Ma [31]
*Robinia pseudoacacia*	B = 0.312 + 0.016D^2^H	He [32]
*Ginkgo biloba*	B = 0.044 + 0.042D^2^H	
*Populus przewalskii*	B = 0.016(D^2^H)^1.007^	Yang [33]
*Ligustrum lucidum*	B = 0.907 + 0.01D^2^H	Hyun-Kil Jo [34]
*Camphora officinarum*	B = 0.937 + 0.037D^2^H	
*Metasequoia glyptostroboides*	B = Exp(−0.8168 + 2.1549log*D*)	
*Pinus thunbergii*	B = 0.1309D^2.4367^	
*Salix babylonica*	B = 0.178D^2.581^	
*Cedrus deodara*	B = 1.26(0.3721D^1.2928^ + 0.2805D^1.3313^)	
*Lagerstroemia indica*	B = 0.895 + 0.035D^2^H	
*Ulmus pumila*	B = 0.1458(D^2^H)0.8191	
Angiosperms	B = 0.0396(D^2^H)^0.933^	Liu [35]
Gymnosperms	B = 0.0254(D^2^H)^0.948^	Wang [36]
*Photinia serratifolia*	B = 0.310(D^2^H)^1.097^	Yao [37]
*Syringa oblata*	B = 6.656H^5.065^	
*Lagerstroemia indica*	B = 11.109 + 17.91ln(H)	
*Pittosporum tobira*	B = 0.264(D^2^H)^0.916^	
*Hibiscus syriacus*	B = 2.958(D^2^H)^0.607^	
*Nandina domestica*	B = 18.925(CH)^1.565^	
*Buxus sinica*	B = 15.572D^1.325^	Yao [38]
*Berberis thunbergii* ‘Atropurpurea’	B = 224.662(CH)^1.546^	
*Euonymus alatus*	B = 68.016(AC)^1.021^	Li [39]
Arborescent Shrub	B = 0.182D^2.487^	
Shrubs	B = 100.71(AC)^0.925^	

#### 2.3.2. Estimation of Green Space Carbon Storage Using Remote Sensing-Based Inversion Methods

Vegetation indices were derived from spectral reflectance values across two or more bands. In this study, five classic and widely validated vegetation indices were selected to construct a regression model for estimating urban green space carbon storage: Normalized Difference Vegetation Index (NDVI), Ratio Vegetation Index (RVI), Difference Vegetation Index (DVI), Soil-Adjusted Vegetation Index (SAVI), and Leaf Chlorophyll Index (LCI). These indices effectively represented both structural and physiological characteristics of urban vegetation and offered robustness against background interference, thereby facilitating broader application and promotion in specific regional contexts.

(7) $$\mathrm{NDVI}=\frac{\mathrm{NIR}-R}{\mathrm{NIR}+R}$$

where R represents the red band reflectance and NI represents the near-infrared band reflectance.

(8) $$\mathrm{RVI}=\frac{\mathrm{NIR}}{R}$$

DVI = NIR − R(9)

(10) $$\mathrm{SAVI}=\frac{\left(\mathrm{NIR}-R\right)\;\left(1+L\right)}{\mathrm{NIR}+R+L}$$

(11) $$\mathrm{LCI}=\frac{B-R}{B+R}$$

where B represents the blue band reflectance.

C = a × X_1_ + b × X_2_ + d (12)

where C represents carbon density, X_1_ and X_2_ denote vegetation indices, and a, b, and d are constant coefficients.

#### 2.3.3. Data Processing and Visualization

Multispectral data were processed using ArcGIS 10.8 to calculate raster-based vegetation indices. IBM SPSS Statistics 24 was used to analyze the significance and correlation of vegetation indices in the regression model, while Origin 2024b was employed for figure generation.

## 3. Results

### 3.1. Vegetation Characteristics of Dongjiakou

#### 3.1.1. Morphological Characteristics of Vegetation

**Figure 3:**
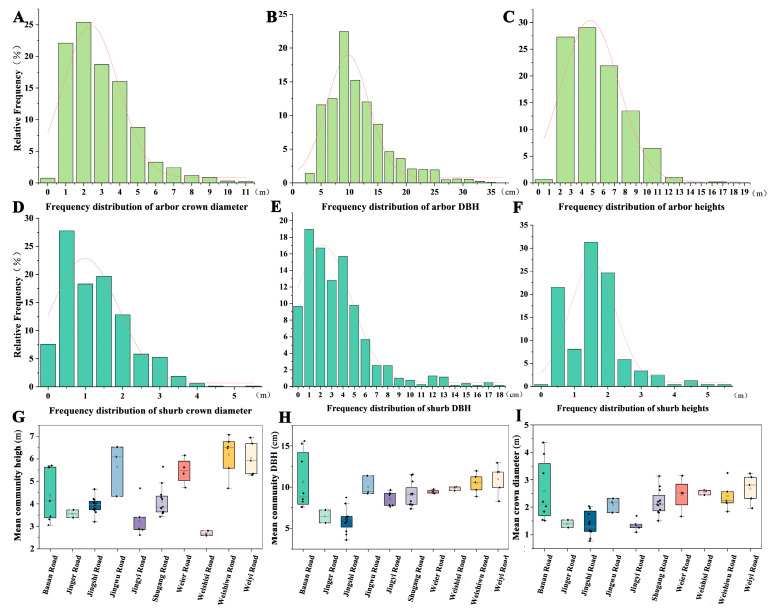
Distribution of vegetation characteristics. Note: (**A**) Freguency distribution of arbor crown diam-eter. (**B**) Frequency distribution of arbor DBH. (**C**) Frequency distribution of arbor heights. (**D**) Frequeney distribution of shurb crown diameter. (**E**) Frequency distribution of shurb DBH. (**F**) Frequeney distribution of shurb heights. (**G**) Mean community heigh of roads. (**H**) Mean community DBH of roads. (**I**) Mean crown diameter of roads.

Field surveys were conducted to statistically analyze the distribution characteristics of all trees and shrubs. The DBH, height, and canopy width of trees followed a left-skewed normal distribution, with most individuals concentrated in smaller size ranges and a few reaching larger dimensions. Tree height ranged from 3 to 9 m (Figure 3A), canopy width varied between 0 and 4.5 m (Figure 3C), and DBH was primarily distributed between 5 and 15 cm, with very few trees exceeding 30 cm (Figure 3B). Shrubs in the port area also exhibited distinct size characteristics. Shrub height was mainly distributed between 1.5 and 2 m (Figure 3D), canopy width ranged from 0.5 to 2 m (Figure 3F), and DBH was within 0 to 8 cm (Figure 3E). These patterns likely reflect the early stage of green space planning and construction in the area, with vegetation not yet fully mature.

Weighted calculations were performed for the average tree height, DBH, and canopy width across different road sections. Significant variations were observed among road segments and within sample plots along the same road. Community average tree height ranged from 2.7 to 6.0 m (Figure 3G), DBH from 6.0 to 11.0 cm (Figure 3H), and canopy width from 1.4 to 2.7 m (Figure 3I). Among all roads, tree height exhibited the highest degree of dispersion compared to DBH and canopy width. Banan Road displayed the greatest variability in all three parameters across sample plots. Notably, the vegetation community on Weishisi Road had an average DBH and canopy width of 9.9 cm and 2.6 m, respectively, indicating moderate levels; however, the average tree height was only 2.7 m, the lowest among all roads. The vegetation community on Weiyi Road had the largest DBH (11.0 cm) and canopy width (2.7 m), with an average tree height of 5.5 m, ranking on the higher end. The highest community average tree height was recorded on Weishiwu Road at 6.2 m, with DBH and canopy width at relatively high levels, measuring 10.5 cm and 2.4 m, respectively.

#### 3.1.2. Spatial Structure Characteristics of Vegetation Communities

Vegetation communities in the study area were classified into four structural types based on vertical stratification: tree-shrub-grass (T-S-G), tree-shrub (T-S), tree-only (T), and shrub-only (S). The stratification of these communities reflected the complexity of both vertical and horizontal structures in green spaces. Survey data indicated that the T-S type was the most prevalent, accounting for 78.57% of all surveyed vegetation communities. The single-layer community structure, including T and S types, was the least common, found in only three sample plots (3.57%). The T-S-G type represented 16.67% of the surveyed vegetation communities (Table 2). These results suggest that vegetation configuration in the port area green spaces primarily emphasizes tree and shrub layers, with an evident lack of herbaceous vegetation.

**Figure 4:**
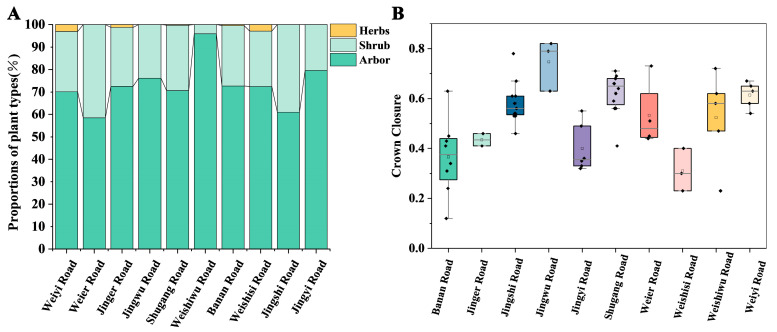
Vegetation community characteristics. Note: (**A**) Proportion of plant types of roads. (**B**) Crown Closure of roads.

The proportion of T-S-G plants reflected the complexity of vertical stratification in the green spaces. In the port area, the current composition of these vegetation layers represented their vertical projection on the total green space area, serving as an indicator of structural complexity. Analyses revealed a significant deficiency in herbaceous vegetation (Figure 4A). Except for Weiyi Road and Weishisi Road, herbaceous coverage remained below 2% across all other roads, with some green spaces entirely lacking herbaceous plants. Among the surveyed roads, Jing Shiwu Road had the highest proportion of trees (75%), while Weishisi Road had the lowest (31%). Banan Road exhibited the highest proportion of shrubs (51%), whereas Jing Shiwu Road had the lowest at only 12%.

The average canopy closure in different vegetation communities of the Dongjiakou green space was 53% (Figure 4B). Most surveyed plots had canopy closure values between 30% and 80%. Jingwu Road had the highest proportion of vegetation communities, with canopy closure exceeding 70%, whereas all sampled plots on Jing’er Road fell within the 30–40% range. Banan Road exhibited the greatest variability, ranging from 0.12 to 0.63. Field observations indicated that, while the overall green coverage on Banan Road was moderate, newly planted young vegetation with small canopies was present in the southwestern section, aligning with the distribution patterns of community average tree height, DBH, and canopy width (Figure 3F–H).

### 3.2. Estimation of Aboveground Carbon Storage in Sample Plots

Carbon storage calculations for each sample plot in Dongjiakou were conducted using field data and allometric biomass equations (Table 1). The results (Table 3) indicated that the average carbon storage density in roadside green spaces was 2.24 kg/m^2^, with significant variations among the 10 surveyed roads. On Weiyi Road, 79.78% of trees were classified as middle-aged or mature, with a dense canopy structure and high values for average tree height, DBH, and canopy width, resulting in the highest carbon density at 4.55 kg/m^2^. In contrast, despite having high canopy closure, tree height, DBH, and canopy width, vegetation on Weishiwu Road exhibited a much lower carbon density of 2.34 kg/m^2^, significantly below that of Weiyi Road. The lowest carbon densities were recorded on Banan Road (1.19 kg/m^2^), Jingshi Road (1.06 kg/m^2^), Shugangyi Road (0.78 kg/m^2^), and Jingyi Road (0.46 kg/m^2^). Notably, Jingshi Road and Shugangyi Road had relatively high canopy closure (Figure 4B), yet their carbon densities remained low. Additionally, Weishiwu Road, Banan Road, Shugangyi Road, and Jingyi Road were all multi-lane roads, which may have influenced vegetation structure and carbon accumulation. In contrast, sample plots on Weishisi Road and Jing’er Road exhibited relatively low canopy closure, tree height, DBH, and canopy width, yet their carbon densities were comparatively high, at 3.74 kg/m^2^ and 3.70 kg/m^2^, respectively. Additionally, Weiyi Road and Jing’er Road were located further from large water bodies (Figure 1).

### 3.3. Estimation of Carbon Density in Dongjiakou

The estimation of carbon sequestration in the Qingdao Port area was conducted using a regression model based on remote sensing parameters and field survey data, a method widely applied in recent years. Sample plot data were used to calculate carbon sequestration values, while multispectral remote sensing data from a DJI Phantom 4 Multispectral UAV collected during the same period were processed to derive vegetation indices reflecting green space growth conditions. A regression model was then developed to estimate the carbon sequestration capacity of existing green spaces in the port area. Vegetation indices, including NDVI, LCI, RVI, SAVI, and DVI, were extracted from sample points and analyzed for correlation with carbon storage and carbon density. SPSS regression analysis indicated that carbon storage and carbon density in Dongjiakou green spaces were significantly correlated with LCI at the 0.05 significance level, while carbon density and DVI exhibited a highly significant correlation at the 0.001 level. Based on the significance of these correlations, LCI and DVI were selected to construct a regression model for estimating carbon density. The final regression equation was as follows:

C = −0.67397 + 0.000972441 × DVI + 9.17439 × LCI

**Figure 5:**
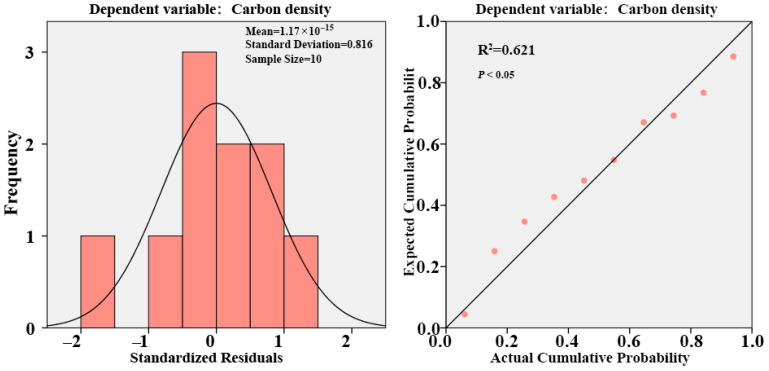
Regression analysis of carbon density in Dongjiakou roadside green spaces.

Regression analyses of different vegetation indices and aboveground carbon storage in Dongjiakou green spaces were performed, considering model correlation and goodness of fit. The final best-fit regression model had an R^2^ value of 0.621, with a statistically significant regression relationship (F-test, *p* < 0.05) (Figure 5).

**Figure 6:**
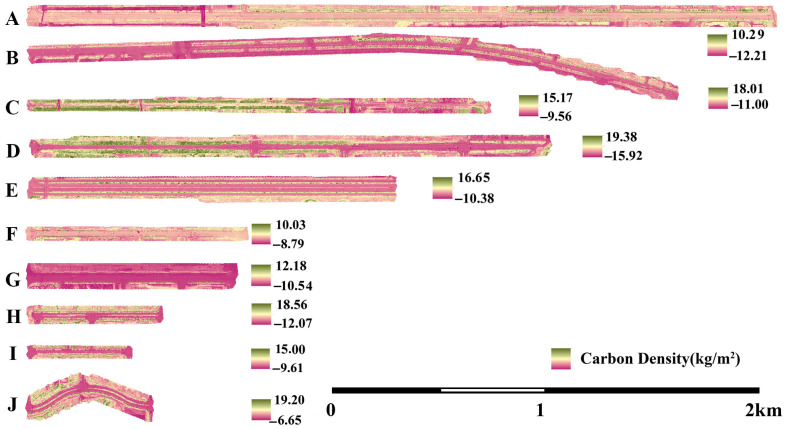
Carbon density distribution in Dongjiakou roadside green spaces. Note: (**A**) Jingshi Road; (**B**) Ba’nan Road; (**C**) Weishiwu Road; (**D**) Weiyi Road; (**E**) Shugangyi Road; (**F**) Weishisi Road; (**G**) Jingyi Road; (**H**) Jing’er Road; (**I**) Wei’er Road; (**J**) Jingwu Road.

Using the regression model, multispectral data from the entire road network were processed to estimate carbon density across all ten surveyed roads through an inversion method. The results are presented in Figure 6. The calculation of grid cells with greater carbon density than zero indicated that the overall road network had an average carbon density ranging from 1.4872 to 4.5581 kg/m^2^. The highest average carbon densities were recorded on Weiyi Road (4.5581 kg/m^2^), Jingwu Road (4.4855 kg/m^2^), Jing’er Road (4.2437 kg/m^2^), and Wei’er Road (3.6064 kg/m^2^). These four roads intersect, forming a high-carbon-density zone. In contrast, the lowest average carbon densities were observed on Weishisi Road (1.4872 kg/m^2^) and Jingyi Road (1.8689 kg/m^2^), both of which are located at a greater perpendicular distance from the shipping center.

### 3.4. Correlation Analysis of Factors Influencing Carbon Density

**Figure 7:**
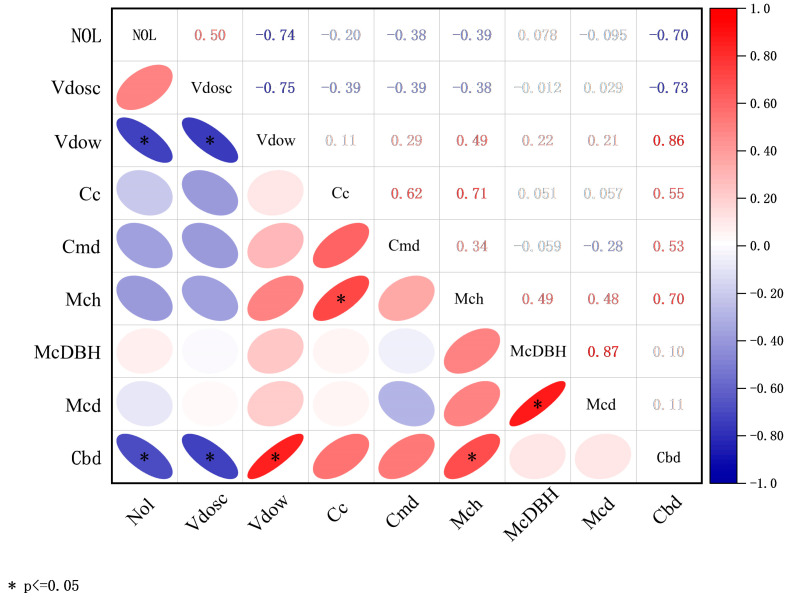
Correlation analysis of factors influencing carbon density. Note: NOL represents the number of lanes; Vdosc represents the distance from the shipping center; Vdow represents the perpendicular distance from major water bodies; Cc represents canopy closure; Cmd represents community density; Mch represents the average height of trees and shrubs in the community; McDBH represents the average DBH of trees and shrubs in the community; Mcd represents the average canopy width of trees and shrubs in the community; Cbd represents carbon density.

The results indicated that significant variations in carbon density existed even among green spaces constructed during the same period. Some roads with high carbon density exhibited considerable differences in vegetation community characteristics, including canopy closure, average tree height, DBH, and canopy width, yet displayed spatial correlations in geographic location. To further investigate these relationships, a correlation analysis was conducted between carbon density and vegetation community characteristics (canopy closure, average tree height, DBH, and canopy width) as well as anthropogenic planning factors (geographic location, road lane number, etc.) (Figure 7). A significant negative correlation was observed between carbon density and the number of road lanes (*p* < 0.05). Similarly, carbon density and the distance from the shipping center showed a significant negative correlation (*p* < 0.05), indicating that carbon density was higher in areas closer to the administrative center of the port, where green space development levels were also higher. Additionally, carbon density and the perpendicular distance from major water bodies exhibited a significant positive correlation (*p* < 0.05), with carbon density increasing as the distance from water bodies increased. Among vegetation community characteristics, only average vegetation height demonstrated a significant positive correlation with carbon density (*p* < 0.05), suggesting that taller vegetation communities generally exhibited higher carbon density.

### 3.5. Estimation of Carbon Storage in Dongjiakou from 2021 to 2024

**Figure 8:**
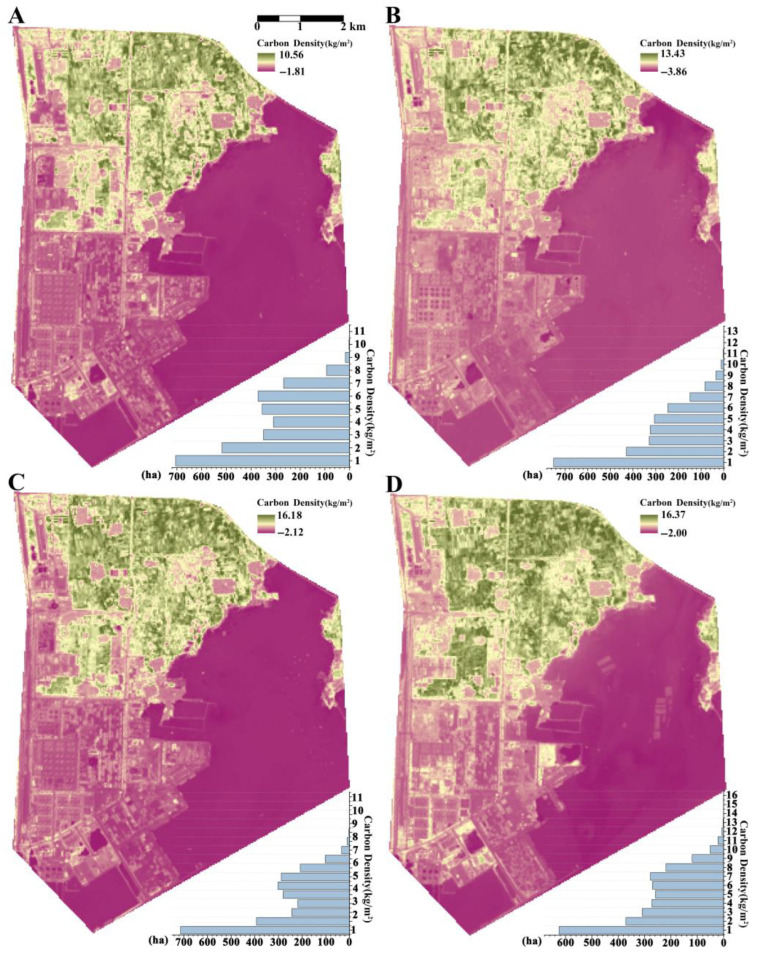
Carbon density distribution in Dongjiakou (2021–2024). Note: Carbon density distribution maps of Dongjiakou Port Area from 2021 to 2024. (**A**) 2021; (**B**) 2022; (**C**) 2023; (**D**) 2024.

Correlation analysis revealed that urban planning and human interventions had a significant impact on vegetation community carbon density. Based on the regression model, the overall carbon density in the Dongjiakou Port area from June 2021 to June 2024 was estimated (Figure 8). The total carbon storage for each year was 108,180.9 t in 2021, 90,815.4 t in 2022, 114,140.7 t in 2023, and 119,238.3 t in 2024. As shown in Table 4, annual carbon sequestration values were −17,365.9 t from 2021 to 2022, 23,325.3 t from 2022 to 2023, and 5097.6 t from 2023 to 2024. Between 2021 and 2023, green space area decreased annually, with losses of 5.22 ha and 131.04 ha, respectively. However, carbon sequestration increased significantly following the 2024 green space enhancement project. A comparison of the 2024 (Figure 8D) and 2023 (Figure 8C) images revealed an increase in the number and extent of green patches. The green space area expanded by 219.68 ha, exceeding the 168.78 ha officially recorded by local authorities, indicating an additional increase of 50.9 ha.

## 4. Discussion

### 4.1. Factors Influencing Carbon Density in Dongjiakou

Field surveys conducted on ten roads in Dongjiakou, all constructed during the same period, revealed significant variations in carbon density. Figure 1 shows that Weiyi Road, Jingwu Road, Jing’er Road, and Wei’er Road, which exhibited the highest carbon density, are geographically close and intersect, and are all located near the shipping center of Qingdao Port Dongjiakou. Correlation analysis indicated that proximity to the port’s administrative center was associated with higher carbon density and more extensive green space development. The strategic significance of the shipping center has led to greater attention to vegetation maintenance and more frequent management along nearby roads, contributing to enhanced carbon sequestration [40]. These findings highlight the substantial influence of anthropogenic factors on urban vegetation carbon sequestration capacity. An increase in the distance from major water bodies corresponded with higher carbon density, a trend consistent with findings by Xu Xu [12] and Chen [41]. The low carbon-dissolving capacity of water bodies influences local atmospheric composition, affecting vegetation carbon sequestration. A greater number of road lanes was correlated with lower vegetation carbon density, reflecting the ecological impact of urbanization and transportation systems. Vehicle emissions and CO_2_ pollution not only degrade air quality but also reduce plant carbon sequestration capacity. Fossil fuel combustion in industrial processes and road transportation remains a primary contributor to urban carbon emissions [42].

Among vegetation community characteristics, only average tree height showed a positive correlation with carbon density. Taller trees with higher canopies gain increased access to sunlight, promoting organic matter synthesis in leaves and enhancing carbon sequestration capacity [43]. However, canopy width, DBH, and canopy closure did not exhibit a significant correlation with carbon density, a result inconsistent with findings by Ahmad [9] and Goodall [44]. This discrepancy may stem from differences in research scale and study areas. Smaller study scales allow for a more detailed analysis of multiple factors influencing carbon density, whereas urban green spaces in Dongjiakou differ from forest ecosystems, making carbon density more susceptible to environmental and anthropogenic influences. For example, Shugangyi Road exhibited a high canopy closure of 0.62 and was dominated by *Cedrus deodara* and *Sabina chinensis*, species known for strong carbon sequestration capacity [45]. However, its carbon density was only 2.25 kg/m^2^, likely due to its location on the periphery of the port, where heavy traffic and multiple lanes contribute to poor vegetation health. Research suggests that vehicle emissions and air pollution negatively affect the carbon sequestration capacity of urban ecosystems [46]. Additionally, the road’s proximity to a large water body may have further influenced its carbon sequestration capacity, highlighting the complex interplay of multiple environmental factors.

### 4.2. Impact of Port Development Planning on Green Spaces in Dongjiakou

The additional 50.9 ha of green space in 2024 may be attributed to ecological restoration under the green space enhancement project, which improved environmental conditions and facilitated natural rewilding. Zoderer [47] and Quintas-Soriano [48] showed that rewilding urban green spaces holds substantial carbon sequestration potential. Green space construction in Dongjiakou Port began in 2020 and was completed in 2021, with newly planted vegetation in its early growth stage, typically exhibiting high carbon sequestration potential. However, rather than increasing, carbon sequestration from 2021 to 2022 declined sharply. This reduction may be linked to major infrastructure developments, including the Dongjiakou Commercial Crude Oil Reserve and Langya Taiwan North Three Wharf, which disrupted the ecological environment and compromised vegetation health, leading to a notable decline in carbon storage. Liu et al. [13] also highlighted the significant impact of land use changes in urbanized areas on carbon sequestration capacity. Despite an overall increase in aboveground carbon storage from 2021 to 2024, following green space enhancement efforts, green areas with carbon density ranging from 3 to 8 kg/m^2^ did not recover after renovation. This finding suggests that the ecological damage caused by large-scale construction projects is not easily reversible in the short term. Additionally, Ren et al. [49] indicated that urban expansion has delayed and long-term ecological consequences, necessitating proactive planning and protective measures in the early stages of development [50].

This paper did not delve deeply into the factors that promote the rewilding of plant communities. Specifically, what factors contribute to the rewilding of plant communities? What are the weights of these ecological indicators in promoting rewilding? This could be a potential direction for future research, allowing us to further explore the influencing factors of urban ecology and promote urban ecological development.

## 5. Conclusions

The carbon density of green spaces in the port area was estimated, and influencing factors were analyzed, leading to the following conclusions:

(1) In the Dongjiakou Port area, carbon density showed a significant positive correlation with average vegetation height, whereas no correlation was observed with other vegetation characteristics. In contrast, carbon density was strongly associated with anthropogenic planning factors.

(2) Infrastructure development had a notable negative impact on vegetation carbon sequestration, with effects not reversible in the short term. However, green space enhancement efforts improved the ecological environment, leading to an additional 50.9 ha of green space. The impact of green space enhancement extended beyond the expansion of constructed green areas, contributing to broader ecological benefits in the port region.

(3) Both urban expansion and green space development have delayed and long-term impacts on the ecological environment. It is recommended that relevant government departments conduct corresponding environmental impact assessments during the planning stage of urban development.

## Figures and Tables

**Table 2:** Proportion of vegetation community vertical structure.

Structure	Vertical Structure Type	Proportion of Sample Plots (%)
Multi-layer Structure	Tree-Shrub-Grass (T-S-G)	16.67%
Two-layer Structure	Tree-Shrub (T-S)	78.57%
Single-layer Structure	Tree (T)	2.38%
	Shrub (S)	1.19%

**Table 3:** Carbon storage in roadside sample plots.

Name	Sample	Dominant Tree Species	Carbon Storage (kg)	Carbon Density (kg/m^2^)
Wei’yi Road	5	*Fraxinus chinensis*, *Platanus acerifolia*, *Pterocarya stenoptera*, *Photinia* × *fraseri*	9099.62	4.55
Wei’er Road	4	*Koelreuteria paniculata*, *Juniperus chinensis* ‘Kaizuca’, *Metasequoia glyptostroboides*, *Euonymus fortunei*,	2987.62	1.87
Jing’er Road	2	*Juniperus chinensis* ‘Kaizuca’, *Cedrus deodara*, *Koelreuteria paniculata*, *Styphnolobium japonicum*, *Buxus sinica* var. *parvifolia*, *Euonymus fortunei*	2960.83	3.70
Jing’wu Road	3	*Juniperus chinensis* ‘Kaizuca’, *Cedrus deodara*, *Styphnolobium japonicum*, *Prunus* × *yedoensis*	3300.03	2.75
Shugang’yi Road	12	*Cedrus deodara*, *Juniperus chinensis* ‘Kaizuca’, *Fraxinus chinensis*, *Hibiscus syriacus*, *Photinia* × *fraseri*	3726.88	0.78
Wei’shiwu Road	5	*Ginkgo biloba*, *Platanus acerifolia*, *Zelkova serrata*, *Malus spectabilis*, *Photinia* × *fraseri*, *Euonymus fortune*	4671.69	2.34
Ban’nan Road	8	*Platanus acerifolia*, *Lagerstroemia indica*, *Photinia* × *fraseri*, *Euonymus fortunei*	3803.39	1.19
Wei’shisi Road	3	*Malus spectabilis*, *Platanus acerifolia*, *Photinia* × *fraseri*	4483.24	3.74
Jing’shi Road	12	*Juniperus chinensis*, *Cedrus deodara*, *Platanus acerifolia*, *Fraxinus chinensis*, *Lagerstroemia indica*, *Cercis chinensis*	5106.69	1.06
Jing’yi Road	6	*Juniperus chinensis* ‘Kaizuca’, *Celtis sinensis*, *Ligustrum japonicum*, *Photinia* × *fraseri*	1098.36	0.46
Toal	60	-	-	2.24

**Table 4:** Changes in aboveground carbon storage in Dongjiakou from 2021 to 2024.

Year	Carbon Storage (t)	Carbon Sequestration (t)	Green Space Area (ha)	Green Space Development Area (ha)	Green Space Area Change (ha)
2021	108,180.9		2803.59		
		−17,365.9		24.1	−5.22
2022	90,815.4		2798.37		
		23,325.3			−131.04
2023	114,140.7		2667.33		
		5097.6		168.78	219.68
2024	119,238.3		2887.01		
		

## Data Availability

Data is contained within the article.
